# Association Between High School Personality Phenotype and Dementia 54 Years Later in Results From a National US Sample

**DOI:** 10.1001/jamapsychiatry.2019.3120

**Published:** 2019-10-16

**Authors:** Benjamin P. Chapman, Alison Huang, Kelly Peters, Elizabeth Horner, Jennifer Manly, David A. Bennett, Susan Lapham

**Affiliations:** 1 Department of Psychiatry, University of Rochester Medical Center, Rochester, New York; 2 Department of Public Health Sciences, University of Rochester Medical Center, Rochester, New York; 3 American Institutes for Research, Washington, DC; 4 Department of Neurology, Columbia University Medical Center, New York, New York; 5 Rush Alzheimer’s Disease Center, Rush University Medical Center, Chicago, Illinois

## Abstract

**Importance:**

Personality phenotype has been associated with subsequent dementia in studies of older adults. However, neuropathologic changes often precede cognitive symptoms by many years and may affect personality itself. Therefore, it is unclear whether supposed dementia-prone personality profiles (high neuroticism and low conscientiousness) are true risk factors or merely reflections of preexisting disease.

**Objectives:**

To examine whether personality during adolescence—a time when preclinical dementia pathology is unlikely to be present—confers risk of dementia in later life and to test whether associations could be accounted for by health factors in adolescence or differed across socioeconomic status (SES).

**Design, Setting, and Participants:**

Cohort study in the United States. Participants were members of Project Talent, a national sample of high school students in 1960. Individuals were identified who received a dementia-associated *International Classification of Diseases*, *Ninth Revision* (*ICD-9*) diagnosis code during any year between 2011 and 2013. The dates of our analysis were March 2018 to May 2019.

**Exposures:**

Ten personality traits were measured by the 150-item Project Talent Personality Inventory. Socioeconomic status was measured by a composite based on parental educational level, income, occupation, and property ownership. Participants were also surveyed on demographic factors and height and weight.

**Main Outcomes and Measures:**

Medicare records were collected, with dementia diagnoses in the period of 2011 to 2013 classified according to the US Centers for Medicare & Medicaid Services *ICD-9*–based algorithm. Cox proportional hazards regression models estimated the relative risk of dementia based on the 10 personality traits, testing interactions with SES and adjusting for demographic confounders.

**Results:**

The sample of 82 232 participants was 50.1% female, with a mean (SD) age of 15.8 (1.7) years at baseline and 69.5 (1.2) years at follow-up. Lower risk of dementia was associated with higher levels of vigor (hazard ratio for 1 SD, 0.93; 95% CI, 0.90-0.97; *P* < .001). Calm and maturity showed protective associations with later dementia that increased with SES. At 1 SD of SES, calm showed a hazard ratio of 0.89 (95% CI, 0.84-0.95; *P* < .001 for the interaction) and maturity showed a hazard ratio of 0.90 (95% CI, 0.85-0.96; *P* = .001 for the interaction).

**Conclusions and Relevance:**

This study’s findings suggest that the adolescent personality traits associated with later-life dementia are similar to those observed in studies of older persons. Moreover, the reduction in dementia risk associated with a calm and mature adolescent phenotype may be greater at higher levels of SES. Personality phenotype may be a true independent risk factor for dementia by age 70 years, preceding it by almost 5 decades and interacting with adolescent socioeconomic conditions.

## Key Points

**Question:**

Are maladaptive personality traits true risk factors for dementia or merely early expressions of underlying neuropathologic changes?

**Findings:**

In a cohort study of 82 232 participants, personality traits in adolescence—a time when dementia pathology is unlikely to be present—were a factor associated with incident dementia almost 5 decades later in a national US cohort. Calm and mature adolescents were less likely to develop dementia, and this risk reduction was significantly more pronounced with higher socioeconomic status.

**Meaning:**

This study’s findings suggest that maladaptive personality traits decades earlier may be independent risk factors for dementia by age 70 years.

## Teaser

This national US cohort study examines whether personality during adolescence—a time when preclinical dementia pathology is unlikely to be present—confers risk of dementia in later life and tests whether associations could be accounted for by health factors in adolescence or differed across socioeconomic status.

## Introduction

Personality phenotype is reportedly associated with incident dementia in older adults.^1,2,3,4,5,6^ One interpretation is that these findings indicate that personality traits are “risk factors” (ie, factors with a role in dementia). However, studies in older samples of patients feature a median follow-up time of 6 years,^1^ and dementia neuropathologic changes can begin several years before symptoms manifest.^7^ Therefore, associations over shorter periods raise the possibility that personality is an early indicator of existing disease rather than a “true” risk factor.

Specifically, a high-neuroticism, low-conscientiousness phenotype appears to predate dementia diagnosis.^8,9^ However, the high-neuroticism, low-conscientiousness profile also matches the pattern of personality deterioration noted during the disease^10^ and sometimes leading up to diagnosis.^11,12^ Furthermore, reduced cerebral gray and white matter volume occurs in some dementias and has been associated cross-sectionally with higher neuroticism and lower conscientiousness in preclinical samples.^13^ These observations suggest that some prospective associations between personality and dementia in old age may reflect early signs of existing disease. Yet, other studies find little evidence of personality change before diagnosis.^14^

Regarding life course timing, familial Alzheimer disease (AD) is a variety of dementia^15^ in which neuropathologic features can develop before age 30 years^16,17^ but is very rare, and in most cases, neuropathologic changes begin no earlier than a few decades before the onset of symptoms.^15^ Therefore, associations between personality in adolescence and later dementia would suggest that personality has some role as a life course risk factor. Moreover, life course models emphasize the role of personality in initiating and maintaining health behaviors, such as physical activity, in the experience of and physiological response to psychological stress and in determining educational achievement^18^ and occupational success.^19^ These outcomes act as mechanisms that, over the decades, build cumulative risk for or protection against dementia.^20,21^ Therefore, we set out to examine whether personality traits measured in high school during 1960 were associated with dementia by age 70 years in a large US sample. We hypothesized that traits associated with the modern Big Five personality dimensions of low neuroticism and high conscientiousness would be associated with lower probability of dementia.^22,23,24^ We also hypothesized that higher socioeconomic status (SES), which confers protection against dementias,^25^ would amplify the protective nature of adaptive adolescent personality traits, while lower SES would dampen such associations.

## Methods

### Participants

Project Talent is a national probability sample of 5% of all US high schools in 1960 (n = 1226).^26^ All students attending these schools (n = 377 016) completed 2 days of tests and questionnaires assessing a variety of abilities, background information, and dispositional factors (ie, personality trait assessments). Recently, a subset of the Project Talent sample (82 232 of 377 016 [21.8%]) was linked to Medicare fee-for-service claims data reflecting a 2011 to 2013 reference period^27^ (eAppendix 1 in the Supplement). Compared with those not in the Medicare analytic sample, those in the Medicare analytic sample were more likely to be of white race by 5%, they showed baseline SES scores about a quarter of a standard deviation higher, and baseline personality differences were small (mean Cohen *d* = 0.09, with a maximum of 0.17)^28^ (eTable 1 in the Supplement).

Informed consent regulations were not established in 1960; therefore, informed consent was not obtained at the base year data collection. The Project Talent–Medicare study was granted a waiver of consent by the American Institutes for Research Institutional Review Board and was approved by the US Centers for Medicare & Medicaid Services (CMS). The study uses an opt-out enrollment model in which participants are notified of the study through regular project communications and informed of how they can opt out should they choose to.

### Project Talent Personality Inventory

The 150-item Project Talent Personality Inventory was designed to measure 10 personality traits with continuous scores ranging from low to high. The scales are named for the “high” end of the dimension they measure (generally adaptive) but effectively measure phenotypes on a spectrum from maladaptive to adaptive. Responses to each item are given on a Likert-type scale. Scale descriptions, Cronbach α internal consistency reliability estimates, and primary Big Five loadings from a validation study^29^ are as follows: sociability (outgoingness and desire for social interaction [high extraversion], 12 items, α = .79), social sensitivity (empathy and sensitivity to others’ feelings [high agreeableness], 9 items, α = .82), impulsivity (recklessness or tendency toward rash action [low conscientiousness combined with high extraversion], 9 items, α = .56), leadership (self-direction and autonomy [high conscientiousness], 5 items, α = .76), vigor (an energetic disposition [high extraversion], 7 items, α = .82), calm (freedom from distressing emotions [low neuroticism], 9 items, α = .85), tidiness (propensity toward organization and order [high conscientiousness], 11 items, α = .85), culture (artistic and intellectual refinement [high openness], 10 items, α = .79), self-confidence (assurance in one’s own judgment and abilities [low neuroticism], 12 items, α = .77), and maturity (responsibility and reliability [high conscientiousness], 25 items, α = .91).

### Outcome

Occurrence of dementia was assessed using the AD and related disorders algorithm developed by the CMS.^30^ The algorithm identifies any individual who receives a dementia-associated *International Classification of Diseases*, *Ninth Revision* (*ICD-9*) diagnosis code (eAppendix 1A in the Supplement) on at least 1 fee-for-service inpatient, skilled nursing facility, home health agency, hospital outpatient, physician, or supplier claim during any year between 2011 and 2013 (the dates of our analysis were March 2018 to May 2019). These diagnoses include AD, frontotemporal dementia, vascular dementia, and other senile dementia *ICD-9* diagnoses. The diagnosis may have been present before the reference period but must have been documented at least once within the reference period. Patients must also have been alive at the start of the reference period, so the classification does not capture very early dementia diagnoses resulting in mortality before the start of records. The CMS also uses a second AD-specific classification algorithm (occurrence of an *ICD-9* code 331.0 during the reference period), which was examined as a secondary outcome. The CMS claims have a sensitivity of 0.85 and a specificity of 0.89 for identification of dementia cases compared with clinical assessment.^31^

### Additional Variables

Additional variables included school grade at baseline, sex, and race (indicators for African American or other minority against a reference category of white race). Family SES was measured by a 9-item index completed by the student, which included parental educational level, income, occupation, housing, and ownership of select appliances indicative in 1960 of financial means, such as washing machines. Details of the index construction can be found in study documentation.^32^ Briefly, items were scored so that greater parental educational level, income, occupational status, and property ownership reflected higher SES, and composite scores were scaled to a mean (SD) of 100 (10). Participants were also surveyed on demographic factors and height and weight. IQ is largely independent of Project Talent Personality Inventory traits (median correlation of 0.09 and maximum of 0.18), and its associations with AD and related disorders in Project Talent have been previously reported.^33^ Self-reported height and weight were used to calculate baseline body mass index (BMI) for supplementary analyses.

### Statistical Analysis

We first examined descriptive statistics of all variables. Multivariable Cox proportional hazards regression models then examined associations between each of the 10 personality traits separately and the dementia end point, adjusted for the demographic confounders of school grade at baseline, sex, race, and SES. Interactions between each personality trait and the SES index were next examined. Tests for main effects and interactions were screened with the false discovery rate.^34^ Continuous variables were scaled to a mean of zero and SD of 1 in regression models so that the hazard ratio (HR) reflects the relative risk associated with a 1-SD increase in the risk factor.

Supplementary analyses (1) examined whether race, in addition to SES, moderated personality associations; (2) assessed whether intercorrelated personality traits that were associated with dementia reflected a common underlying factor and, if so, whether the underlying factor was a risk factor for dementia; (3) adjusted for BMI as a proxy for early cardiovascular health and physical activity; and (4) repeated models with the AD-specific CMS classification. Sensitivity analyses compared personality trait relative risks with and without accounting for selection into the analytic sample using Heckman selection models with traits, race, and baseline SES in the selection equation. Additional sensitivity analyses probed the potential result of both random and systematic outcome misclassification using simulation methods^35^ similar to those of a prior dementia study^36^ with similar design, and they examined whether any adolescent personality trait was a risk factor associated with use of health services where a dementia diagnosis might be rendered.

Tests were 2 sided, with the false discovery rate maintained at .05. Analyses were conducted in Stata (versions 14 and 15; StataCorp LLC).

## Results

### Main Effects

Table 1 lists participant characteristics for this cohort study in the United States overall and stratified by dementia outcome. Among 82 232 participants (41 230 [50.1%] of whom were female), the mean (SD) age was 15.8 (1.7) years at baseline and 69.5 (1.2) years at follow-up, an average span of 53.7 years. Personality traits were approximately symmetrically distributed, with some excess kurtosis. Of 82 232 individuals, 2543 (3.1%) met dementia criteria during the index period. Model specification tests revealed no evidence of nonproportional or school grade–specific hazards for personality traits. The false discovery rate rejection threshold across all tests of main effects and interactions was 0.00125.

**Table 1.** Sample Description Overall and by Dementia Outcome

Variable	Overall (N = 82 232)	Noncases (n = 79 689)	Cases (n = 2543)
School grade at baseline, sex, and race, No. (%)			
Freshman	21 713 (26.4)	21 201 (26.6)	512 (20.1)
Sophomore	21 808 (26.5)	21 217 (26.6)	591 (23.2)
Junior	19 258 (23.4)	18 601 (23.3)	657 (25.8)
Senior	19 453 (23.7)	18 670 (23.4)	783 (30.8)
Male	41 002 (49.9)	39 818 (50.0)	1184 (46.6)
Female	41 230 (50.1)	39 871 (50.0)	1359 (53.4)
African American race	2713 (3.3)	2565 (3.2)	148 (5.8)
Other minority	2385 (2.9)	2314 (2.9)	71 (2.8)
White race	76 530 (93.1)	74 214 (93.1)	2316 (91.1)
Age and personality traits, mean (SD)^a^			
Age, y	69.51 (1.23)	69.50 (1.23)	69.84 (1.22)
SES composite	99.7 (9.7)	99.8 (9.7)	98.9 (9.8)
Sociability	6.73 (2.93)	6.74 (2.93)	6.64 (2.94)
Social sensitivity	4.75 (2.37)	4.75 (2.37)	4.84 (2.37)
Impulsivity	1.94 (1.64)	1.93 (1.64)	2.01 (1.66)
Leadership	1.32 (1.39)	1.32 (1.39)	1.34 (1.37)
Vigor	3.76 (2.16)	3.77 (2.16)	3.58 (2.17)
Calm	4.44 (2.54)	4.44 (2.54)	4.34 (2.56)
Tidiness	5.84 (2.84)	5.84 (2.84)	5.80 (2.84)
Culture	5.32 (2.39)	5.32 (2.39)	5.31 (2.39)
Self-confidence	1.32 (1.39)	1.32 (1.39)	1.34 (1.37)
Maturity	5.22 (2.52)	5.23 (2.52)	5.09 (2.57)
General personality factor	11.63 (5.33)	11.64 (5.33)	11.50 (5.26)

Abbreviation: SES, socioeconomic status.

^a^ Personality traits are in raw score metric. The SES composite is scaled so that the mean (SD) is 100 (10).

The top part of Table 2 lists covariate-adjusted personality trait HRs in main effects models. Vigor exhibited a significant unmoderated association with later dementia (HR for 1 SD, 0.93; 95% CI, 0.90-0.97; *P* < .001). As an effect size benchmark, this 7% risk reduction was equivalent to the risk reduction for 1 SD of SES in main effects models (eTable 2 in the Supplement). Among the covariates in the models, higher dementia risk was also associated with being older in 1960 (HR, 1.75; 95% CI, 1.57-1.96; *P* < .001 for seniors vs freshmen) and with being of African American race compared with white race (HR, 1.69; 95% CI, 1.40-2.00; *P* < .001). Female risk was elevated relative to male risk (HR, 1.12; 95% CI, 1.04-1.21; *P* < .001). eTable 2 in the Supplement lists the full sets of model parameter estimates.

**Table 2.** Adolescent Personality Trait Associations With Later-Life Dementia Risk Among 82 232 Participants^a^

Variable	HR (95% CI)	*P* Value
**Main Effects**^**b**^		
Sociability	0.96 (0.92-1.00)	.046
Social sensitivity	1.01 (0.97-1.05)	.62
Impulsivity	1.04 (1.00-1.08)	.03
Leadership	1.02 (0.98-1.06)	.43
Vigor	0.93 (0.90-0.97)	<.001
Calm	0.95 (0.91-0.99)	.007
Tidiness	0.97 (0.93-1.01)	.08
Culture	0.97 (0.93-1.01)	.11
Self-confidence	0.94 (0.91-0.98)	.004
Maturity	0.96 (0.92-1.00)	.03
**Moderated Associations**^**c**^		
Calm		
Low SES, −1 SD	1.02 (0.97-1.08)	.46
Average SES, mean	0.95 (0.92-0.99)	.02
High SES, 1 SD	0.89 (0.84-0.95)	<.001
Maturity		
Low SES, −1 SD	1.04 (0.98-1.09)	.17
Average SES, mean	0.97 (0.93-1.00)	.11
High SES, 1 SD	0.90 (0.85-0.96)	.001

Abbreviations: HR, hazard ratio; SES, socioeconomic status.

^a^ Hazard ratios (95% CIs) are from Cox proportional hazards regression models adjusted for school grade at baseline, sex, race (indicators for African American race, other minority, and missing race against a reference category of white race), and SES. The false discovery rate rejection threshold across all tests of main effects and interactions is 0.00125. Full regression tables showing covariate associations are presented in eAppendix 2 in the Supplement.

^b^ The top part of the table shows main effects from models with no interaction terms. Significant interactions with SES included calm and maturity, with HRs of 0.94 (95% CI, 0.90-0.97; *P* = .001) for calm interaction term and 0.93 (95% CI, 0.90-0.97; *P* < .001) for maturity interaction term.

^c^ The bottom part of the table shows HRs for calm and maturity at low, medium, and high SES.

### Personality by SES Interactions

Interaction tests indicated that SES significantly moderated the association of both calm and maturity with later dementia diagnosis. The bottom part of Table 2 lists the HRs and 95% CIs for calm and maturity at SES levels of −1 SD, the mean, and 1 SD. At higher SES, a 1-SD increase in calm exhibited an HR of 0.89 (95% CI, 0.84-0.95; *P* < .001 for the interaction), whereas the same increase in maturity was associated with an HR of 0.90 (95% CI, 0.85-0.96; *P* = .001 for the interaction). At lower SES, neither personality trait reduced dementia risk (HRs of 1 or slightly higher at SES levels of −1 SD).

### Supplementary Analyses

No significant interactions between race and personality were noted. Factor analysis of the Project Talent Personality Inventory Scales indicated a general factor defined primarily by high loadings (ie, >0.6 to 0.7) of social sensitivity, calm, tidiness, culture, and maturity (eTable 3 in the Supplement). This “general personality factor” reflected a globally adaptive adolescent phenotype that showed a protective association against later-life dementia similar to its constituent personality traits (eTable 4 in the Supplement), with more pronounced risk reduction at higher SES (eFigure 1 in the Supplement). Additional adjustment for adolescent BMI showed that BMI was associated with increased dementia risk (1-SD BMI HR, 1.07; 95% CI, 1.03-1.11; *P* < .001), but vigor remained strongly protective (HR, 0.94; 95% CI, 0.90-0.98; *P* < .001).

A final set of supplementary analyses repeated all models with the CMS AD classification (865 cases). Personality trait associations were consistent with those seen for AD and related disorders, falling within the 95% CIs of those estimates (eTable 5 in the Supplement). Main effects HRs were 0.90 (95% CI, 0.84-0.96) for vigor, 0.93 (95% CI, 0.84-0.96) for calm, and 0.91 (95% CI, 0.85-0.97) for maturity. Interaction terms were virtually identical in point estimates (ie, 0.94 vs 0.95 for calm and 0.93 vs 0.94 for maturity), and all estimates showed slightly wider 95% CIs.

### Sensitivity Analyses

Models accounting for selection into the Medicare sample based on race and SES revealed estimates for personality trait relative risks similar to those in the primary models (eAppendix 3 and eTable 6 in the Supplement). Random deviation of up to 10% around the published 0.86 CMS algorithm sensitivity resulted in minimal relative risk change (<0.01) (eFigure 2 in the Supplement). Higher diagnostic sensitivity among those who were lower in a protective personality trait, such as calm or maturity, attenuated the relative risk, whereas higher sensitivity in those who were higher in a protective personality trait amplified it (10% sensitivity differences altering the 0.90 to 0.94 and 0.87, respectively) (eFigure 3 in the Supplement). Finally, vigor, calm, and maturity among undiagnosed persons did not lead to greater use of health services where CMS dementia diagnoses were rendered (eTable 7 in the Supplement).

## Discussion

In this study, vigor, calm, and maturity in adolescence were associated with lower risk of dementia roughly 54 years later. A 1-SD increase in vigor reduced later-life dementia risk by about 7%, approximately the same as a 1-SD increase in SES. Vigor is within the extraversion domain of the Big Five personality taxonomy and reflects vitality, energy, and general activity level. Evidence suggests at least some continuity of physical activity from adolescence to adulthood.^37^ A study^38^ in middle-aged and older adults found a large but nonsignificant protective association between dementia and the similar personality trait of “activity,” which is a disposition toward busier lifestyles^39^ and physical activity.^40^ Although higher adolescent BMI was also associated with greater dementia risk, it did not account for the vigor association. Body mass index is an imperfect proxy for physical activity, and some items in the vigor scale refer to more general markers of enthusiastic life engagement (ie, “I am full of pep and energy” or “People seem to think I lead a vigorous life”). These features of the phenotype could extend beyond physical forms of activity to other qualities, such as purpose in life^41^ and/or social engagement.^42,43^ Socioeconomic status also did not moderate this personality trait. While social class may shape the specific type or content of one’s life activities, the beneficial nature of energetic enthusiasm appears to be constant across the SES spectrum.

Calm and maturity showed protective associations with later dementia in a manner dependent on adolescent SES. As SES decreased, the protective associations of these personality traits eroded: at low SES (−1 SD), both traits were essentially not associated with later dementia. Some protective associations were noted at average SES: at a level of SES 1 SD above the mean, adolescent calm and maturity were each associated with roughly a 10% reduction in dementia risk. When their correlation was accounted for by using a single factor largely defined by these 2 personality traits, identical results were noted.

Calm is an indicator of low levels of Big Five neuroticism,^30^ many facets of which are pronounced near-term risk factors for dementia in older persons.^38^ Explanations for these associations often involve physiological responses to chronic stress, such as dysregulation of the hypothalamic-pituitary-adrenal axis, leading to ongoing glucocorticoid activity.^5,44^ The SES gradient itself is a proxy of exposure to chronic stress arising from everyday social environments rife with challenges.^45^ Features of lower adolescent SES, such as financial stress, transportation issues, and housing problems as well as increased exposure to crime, may effectively negate the benefits of a calm disposition on stress response pathways implicated in dementia. Lower earlier-life SES often persists for many,^46^ producing an accumulation of social-environmental risks.^47^ Maturity reflects task and goal orientation, reliability, and responsibility, features of the Big Five domain of conscientiousness.^29^ Later-life conscientiousness also appears to be protective against dementia.^1,38^ Higher SES in adolescence likely presents greater opportunities for reliability and responsibility to produce long-term benefits from higher educational and occupational status, which enhance cognitive reserve.

Because dementia pathology is absent during adolescence in most instances, these findings suggest that personality phenotype may function as a risk factor for dementia. However, it is important to note that risk factor vs early indicator explanations are not mutually exclusive. This complex picture warrants nuance in how reported associations between personality traits and dementias are understood and interpreted clinically. It also questions whether distinct personality signatures characterize the preexisting risk factor/early prodrome/progressive degeneration phases or whether there is simply a common theme throughout these stages. For instance, it is possible that persons who early in life display high levels of neuroticism-related traits and low levels of conscientiousness-related traits pursue a life involving higher dementia risk, who then enter an incipient period of early disease in which neuroticism rises and conscientiousness declines in conjunction with the advent of cognitive symptoms. Although clinical assessment usually focuses on recent personality change, it may also be useful to inquire about personality characteristics much earlier in life.

### Strengths and Limitations

Our study has strengths and limitations. While other studies^33,48,49,50^ have shown links between early-life cognitive abilities and later dementia, the present study is the first to our knowledge to do so for personality traits. Our follow-up extended more than 50 years; however, our sample has not yet reached the ages of the mid-70s and mid-80s. Therefore, cases identified over the index period likely represent a mixture of early onset both before and after the age of 65 years, a common demarcation for early-onset AD vs later-onset AD, which appear to differ in clinical characteristics, genetic risk, and other aspects of disease.^51,52^ However, earlier-onset cases of AD in our sample do not include those diagnosed so early that they would have been deceased before the index period (ie, the rare cases diagnosed in their 40s or 50s and deceased before age 65 years). Premature censoring based on the outcome in question, even if rare, potentially may lead to estimates biased in an unknown direction and to an unknown degree. When differences between the Medicare subsample and other portions of Project Talent were accounted for, sample selection models suggested comparable associations. Selection mechanisms may include early mortality, and work in other portions of the cohort suggests that low levels of adolescent vigor, calm, and maturity increased all-cause mortality risk.^53^ Furthermore, it was not possible to more finely parse different forms or stages of dementia in our study. Because cases must also have come to the attention of medical providers, patients who do not seek care and/or have subclinical forms of cognitive impairment were not captured. In addition, diagnostic biases related to physician or family characteristics could potentially alter classification in unknown ways. This study was also not a study of life course mechanisms; therefore, we did not test specific pathways, although such an aim might be pursued in future work.

## Conclusions

Ultimately, the findings herein underscore the importance of considering earlier-life social circumstances and personality in evaluating dementia risk in addition to more recent information. Personality phenotype may be a true independent risk factor for dementia by age 70 years, preceding it by almost 5 decades and interacting with adolescent socioeconomic conditions.

## Notes

Supplement. **eAppendix 1.** Medicare Linkage Information **eAppendix 2.** Supplementary Analyses **eAppendix 3.** Sensitivity Analysis **eTable 1.** Differences Between Those Included and Not Included in the Medicare Analytic Sample **eTable 2.** Associations Between Personality Traits and CMS ADRD Diagnosis **eTable 3.** Trait Loadings on General Personality Factor and Intercorrelations **eTable 4.** General Personality Factor Associations With Dementia **eTable 5.** Associations Between Personality Traits and CMS AD Classification **eTable 6.** Comparison of Medicare Analytic Sample Model vs Selection Model Results **eTable 7.** High School Personality Traits and Service Use Across Medicare Index Period **eFigure 1.** Association of General Personality Factor With Dementia Risk at Varying SES **eFigure 2.** Impact of Nondifferential Misclassification on Log Relative Risk and Standard Error **eFigure 3.** Impact of Differential Misclassification on Log Relative Risks **eReferences.**
