# Supplementary material for: Association Between High School Personality Phenotype and Dementia 54 Years Later in Results From a National US Sample
Source: JAMA Psychiatry. 2019 Oct 16;77(2):148–54. doi: 10.1001/jamapsychiatry.2019.3120 (PMC6802373; doi:10.1001/jamapsychiatry.2019.3120)
Supplement: Supplement. — eAppendix 1. Medicare Linkage Information eAppendix 2. Supplementary Analyses eAppendix 3. Sensitivity Analysis eTable 1. Differences Between Those Included and Not Included in the Medicare Analytic Sample eTable 2. Associations Between Personality Traits and CMS ADRD Diagnosis eTable 3. Trait Loadings on General Personality Factor and Intercorrelations eTable 4. General Personality Factor Associations With Dementia eTable 5. Associations Between Personality Traits and CMS AD Classification eTable 6. Comparison of Medicare Analytic Sample Model vs Selection Model Results eTable 7. High School Personality Traits and Service Use Across Medicare Index Period eFigure 1. Association of General Personality Factor With Dementia Risk at Varying SES eFigure 2. Impact of Nondifferential Misclassification on Log Relative Risk and Standard Error eFigure 3. Impact of Differential Misclassification on Log Relative Risks eReferences. [file jamapsychiatry-77-148-s001.pdf]

Supplementary Online Content

Chapman BP, Huang A, Peters K, et al. Association between high school personality phenotype and dementia 54 years later in results from a national US sample. *JAMA Psychiatry*. Published online October 16, 2019. doi:10.1001/jamapsychiatry.2019.3120

eAppendix 1. Medicare Linkage Information

eAppendix 2. Supplementary Analyses

eAppendix 3. Sensitivity Analysis

eTable 1. Differences Between Those Included and Not Included in the Medicare Analytic Sample

eTable 2. Associations Between Personality Traits and CMS ADRD Diagnosis

eTable 3. Trait Loadings on General Personality Factor and Intercorrelations

eTable 4. General Personality Factor Associations With Dementia

eTable 5. Associations Between Personality Traits and CMS AD Classification

eTable 6. Comparison of Medicare Analytic Sample Model vs Selection Model Results

eTable 7. High School Personality Traits and Service Use Across Medicare Index Period

eFigure 1. Association of General Personality Factor With Dementia Risk at Varying SES

eFigure 2. Impact of Nondifferential Misclassification on Log Relative Risk and Standard Error

eFigure 3. Impact of Differential Misclassification on Log Relative Risks

eReferences.

This supplementary material has been provided by the authors to give readers additional information about their work.

© 2019 Chapman BP et al. *JAMA Psychiatry.*

eAppendix 1. Medicare Linkage Information

1A. Alzheimer's Disease and Related Disorders (ADRD) Algorithm

The following ICD-9 codes are used by the Centers for Medicare and Medicaid Services (CMS) to identify ADRD cases: 331.0, 331.11, 331.19, 331.2, 331.7, 290.0, 290.10, 290.11, 290.12, 290.13, 290.20, 290.21, 290.3, 290.40, 290.41, 290.42, 290.43, 294.0, 294.10, 294.11, 294.20, 294.21, 294.8, and 797.

1B. Matching procedures.

In 2016, American Institutes for Research submitted 199,994 unique Project Talent cases for matching with Medicare records and claims data. CMS uses Social Security Number or a combination of last name, date of birth, and sex to match cases to Medicare records. Social Security Numbers were collected during Project Talent follow-up data collections and were available for 137,396 unique cases. Given the limited number of variables used in the matching algorithm, multiple aliases were submitted for unique cases to maximize the match rate.

1C. Matching Results.

CMS returned matches for 145,183 individuals. All matches were reviewed for accuracy by comparing name, address, and demographics in Project Talent records to those provided in the matched Medicare record and 99% were accepted as true matches. American Institutes for Research rejected 1,485 matched cases due to incongruent information across the Project Talent data files and the Medicare records.

Returned data include Medicare Parts A, B, and C data. Parts A and B involve fee-for-service data for which reporting to CMS is mandatory. Part C involves data that is reported only voluntarily, and hence often missing. For this reason, the Research Data Assistance Center recommends that Part C data not be used in analyses[1]. Of the 143,698 accepted matched cases, 82,232 had Part A / B data for all of 2012 and 2013, and had complete Project Talent Base Year data for the variables of interest, and thus were included in the present analysis. In comparing those with Parts A and B vs. Part C data, no differences in personality factors exceeding .1 standard deviation SD were noted. Part C individuals were roughly 3 percent more likely to be female 53.2% vs. 49.9%.

1D. Differences Between Medicare Analytic Sample and Others.

We next examined whether individuals in the analytic sample (Medicare Subsample) differed from those excluded from the sample (Non-Medicare) on demographic and personality factors. Any differences could be due to a wide range of factors, including not being in the sampling frame, death before the beginning of the 3-year index period prior to 2011, or not having Medicare Part A / B data.

© 2019 Chapman BP et al. *JAMA Psychiatry.*

eTable 1. Differences Between Those Included and Not Included in the Medicare Analytic Sample

	Medicare Subsample		Non-Medicare		Total				
	n=82,232		n=294,784		n=377,016				
	Frequency	Percent	Frequency	Percent	Frequency	Percent	χ2	p	% Difference
Grade in which student was enrolled at time of testing									
9	21713	26.41%	82180	27.88%	103893	27.56%			-1.44
10	21808	26.52%	77765	26.38%	99573	26.41%			.14
11	19258	23.42%	73161	24.82%	92419	24.51%			-1.4
12	19453	23.66%	61677	20.92%	81130	21.52%			2.74
Unknown	0	0.00%	1	0.00%	1	0.00%			
							326.03	<.001	
Race									
White	77857	94.68%	260159	88.25%	338016	89.66%			6.43
Black	2501	3.04%	24509	8.31%	27010	7.16%			-5.27
Other	1874	2.28%	10116	3.43%	11990	3.18%			-1.15
							3059.96	<.001	
Sex									
Male	41050	49.92%	147124	49.91%	188174	49.91%			-.01
Female	41182	50.08%	147659	50.09%	188841	50.09%			.01
Unknown	0	0.00%	1	0.00%	1	0.00%			
							0.28	0.87	
	Mean	Std. Dev.	Mean	Std. Dev.			t	p	Cohen's D
Age	69.51	1.23	69.62	1.25			23.58	<.001	0.09
SES and Personality / Score Range									

© 2019 Chapman BP et al. *JAMA Psychiatry*.

SES / 58-131	99.75	9.70	97.13	10.28			-68.23	<.001	-.26
Sociability / 0-12	6.73	2.93	6.49	2.94			-20.72	<.001	-0.08
Social Sensitivity / 0-9	4.75	2.37	4.50	2.35			-27.61	<.001	-0.11
Impulsive / 0-9	1.94	1.64	1.94	1.63			0.63	0.53	0.00
Vigor / 0-7	3.76	2.16	3.53	2.11			-27.58	<.001	-0.11
Calm / 0-9	4.44	2.54	4.06	2.49			-37.68	<.001	-0.15
Tidy / 0-11	5.84	2.84	5.46	2.79			-33.74	<.001	-0.13
Culture / 0-10	5.32	2.39	5.04	2.36			-29.17	<.001	-0.12
Leadership / 0-5	1.32	1.39	1.27	1.34			-8.62	<.001	-0.03
Self Confidence / 0-12	5.22	2.52	4.97	2.44			-25.61	<.001	-0.10
Maturity / 0-24	11.63	5.33	10.73	5.14			-43.94	<.001	-0.17

Notes: N = 82,232 in Medicare subsample. 294,784 not in Medicare subsample. Cohen's D reflects the difference in continuous variables in standard deviation units.

© 2019 Chapman BP et al. *JAMA Psychiatry*.

eAppendix 2. Supplementary Analyses

2A: Full Main Effects Regression Table

The following table shows all parameter estimates HRs and 95% confidence intervals for covariate-adjusted Cox proportional hazard models assessing the main effects of personality traits.

© 2019 Chapman BP et al. *JAMA Psychiatry.*

eTable 2. Associations Between Personality Traits and CMS ADRD Diagnosis

	Sociabil-Ity Model	Social Sensitivity Model	Impuls-Ivity Model	Vigor Model	Calm Model	Tidiness Model	Culture Model	Leader-Ship Model	Self-Confidence Model	Maturity Model
Sociability	0.96*									
	0.92, 1.00									
Social Sensitivity		1.01								
		0.97, 1.05								
Impulsivity			1.04*							
			1.00, 1.08							
Vigor				0.93***						
				0.90, 0.97						
Calm					0.95**					
					0.91, 0.99					
Tidiness						0.97				
						0.93, 1.01				
Culture							0.97			
							0.93, 1.01			
Leadership								1.02		
								0.98, 1.06		
Self-Confidence									0.94**	
									0.91, 0.98	
Maturity										0.96*
										0.92, 1.0
Sophomore	1.15*	1.15*	1.15*	1.15*	1.16*	1.15*	1.156	1.15*	1.15*	1.16*
	1.03 - 1.30	1.02 - 1.29	1.02 - 1.29	1.02 - 1.30	1.03, 1.30	1.03, 1.30	1.03, 1.30	1.02, 1.30	1.03, 1.30	1.03, 1.30
Junior	1.45***	1.44***	1.44***	1.45***	1.46***	1.45***	1.46***	1.45***	1.46***	1.46***
	1.30, 1.63	1.29, 1.62	1.29, 1.62	1.29, 1.63	1.30, 1.64	1.30, 1.63	1.30, 1.64	1.29, 1.62	1.30, 1.64	1.30, 1.64
Senior	1.73***	1.71***	1.71***	1.72***	1.75***	1.73***	1.73***	1.72***	1.74***	1.74***
	1.54, 1.93	1.53, 1.92	1.53, 1.91	1.54, 1.93	1.56, 1.96	1.55, 1.94	1.55, 1.94	1.53, 1.92	1.55, 1.94	1.56, 1.95
Female	1.12**	1.12**	1.12**	1.12**	1.121**	1.122*	1.12**	1.12**	1.12**	1.12**

© 2019 Chapman BP et al. *JAMA Psychiatry*.

	1.04, 1.21	1.04, 1.21	1.04, 1.21	1.04, 1.21	1.04, 1.21	1.04, 1.21	1.04, 1.21	1.04, 1.21	1.04, 1.21	1.04, 1.21
African American	1.70***	1.68***	1.68***	1.68***	1.69***	1.68***	1.69***	1.67***	1.69***	1.68***
	1.41, 1.98	1.41, 1.99	1.41, 1.99	1.41, 2.0	1.42, 2.00	1.42, 2.00	1.42, 2.00	1.40, 1.98	1.43, 2.01	1.42, 1.99
Other Minority	0.95	0.95	0.96	0.94	0.95	0.95	0.95	0.95	0.95	0.95
	0.75, 1.20	0.75, 1.21	0.76, 1.21	0.74, 1.19	0.75, 1.20	0.75, 1.21	0.75, 1.21	0.75, 1.21	0.75, 1.21	0.75, 1.20
Missing Race	0.56	0.57	0.57	0.57	0.57	0.57	0.57	0.57	0.57	0.57
	0.28, 1.13	0.28, 1.14	0.285 - 1.15	0.282 - 1.136	0.28, 1.14	0.28, 1.14	0.28, 1.14	0.28, 1.14	0.28, 1.14	0.29, 1.15
SES	0.92***	0.92***	0.92***	0.93***	0.93***	0.92***	0.93***	0.92***	0.93***	0.93***
	0.89, 0.96	0.88, 0.96	0.88, 0.95	0.89, 0.97	0.89, 0.97	0.88, 0.96	0.89, 0.97	0.88, 0.96	0.89, 0.97	0.89, 0.97

Notes: Hazard Ratios in top cell, 95% Confidence interval in bottom cell. N = 82,232 in all models. * = p < .05, ** = p < .01, *** = p < .001. For high school grade, Freshman is reference category; for sex, Male is reference category; for race, White is reference category.

© 2019 Chapman BP et al. *JAMA Psychiatry*.

2C: Personality Factor Analysis

As a whole the marginal distributions of PTPI scales were symmetric (median skew .08, range -.23 - .9) and somewhat leptokurtotic (median kurtosis 2.27, range 1.85 – 3.68), and their joint distribution was approximated by a multivariate normal density (correlations seen in eTable 3, next page). The correlation matrix was subjected to an exploratory factor analysis to determine if one or more broader factors accounted for their intercorrelations. The first and second unrotated Eigenvalues were 4.04 and 0.36, respectively, indicating a dominant first factor. This factor appeared to correspond to the so-called “general personality factor” (GPF), reflecting a collection of related adaptive personality characteristics forming a general dimension. eTable 3 below shows the loadings of the PTPI scale on this factor. Factor scores were estimated via Bartlett’s method, and used as a regressor with the same set of covariates as in other models. eTable 4 shows the full regression model of its associations with dementia. Coefficients reflect the association of 1 SD change in the GPF, and the model includes the interaction with SES. The plot of the association between the GPF and dementia risk at -1 SD, mean levels, and +1 SD SES is shown in eFigure 1.

© 2019 Chapman BP et al. *JAMA Psychiatry.*

eTable 3. Trait Loadings on General Personality Factor and Intercorrelations

	Factor Loading	Sociability	Social Sensitivity	Impulsivity	Vigor	Calm	Tidiness	Culture	Leadership	Self-Confidence	Maturity
Sociability	0.64										
Social Sensitivity	0.79	0.50									
Impulsivity	0.25	0.22	0.19	1.00							
Vigor	0.63	0.49	0.39	0.23	1.00						
Calm	0.76	0.40	0.56	0.12	0.10	1.00					
Tidiness	0.73	0.38	0.51	0.07	0.37	0.50	1.00				
Culture	0.79	0.42	0.61	0.16	0.38	0.51	0.58	1.00			
Leadership	0.61	0.37	0.40	0.24	0.40	0.38	0.32	0.41	1.00		
Self-Confidence	0.50	0.36	0.27	0.11	0.31	0.42	0.26	0.29	0.32	1.00	
Maturity	0.84	0.37	0.56	0.16	0.48	0.59	0.60	0.57	0.48	0.40	1.00

Notes: Results from a principal axis factor analysis of PTPI scales, N = 82, 232. First column is factor loadings.

© 2019 Chapman BP et al. *JAMA Psychiatry*.

eTable 4. General Personality Factor Associations With Dementia

Predictor	HR / 95% CI
General Personality Factor	0.96
	0.93, 1.00
General Personality*SES	0.94***
	0.90, 0.98
SES	0.93***
	0.89, 0.97
Sophomore	1.16*
	1.03, 1.30
Junior	1.46***
	1.30, 1.64
Senior	1.74**
	1.55, 1.94
Female	1.14**
	1.05, 1.23
African American	1.68***
	1.42, 2.0
Other Minority	0.96
	0.76, 1.2
Missing Race	0.57
	0.28, 1.14

Notes: Hazard Ratios in top cell, 95% Confidence interval in bottom cell. N = 82,232 in all models. * = p < .05, ** = p < .01, *** = p < .001.

© 2019 Chapman BP et al. *JAMA Psychiatry.*

eFigure 1. Association of General Personality Factor With Dementia Risk at Varying SES

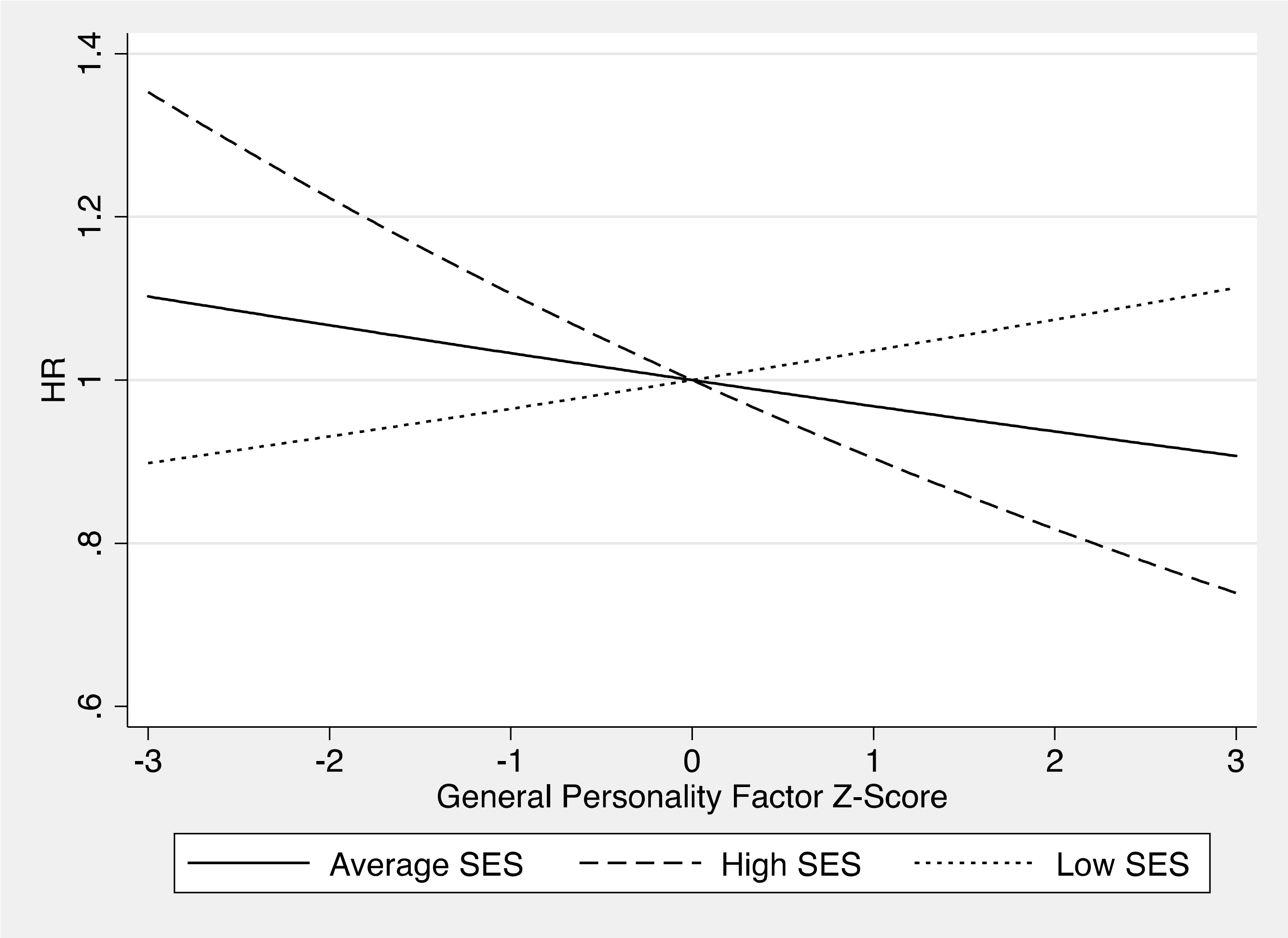

eFigure 1 Caption: Slopes at -1 SD, the mean, and +1 SD SES, based on model in eTable 4.

© 2019 Chapman BP et al. *JAMA Psychiatry.*

2D: Associations with CMS Alzheimer's Disease Outcome

In addition to the primary outcome, based on the CMS algorithm for Alzheimer's Disease and Related Disorders, we also examined as a secondary outcome the CMS algorithm for Alzheimer's Disease only. This involved receiving an ICD-9 diagnosis of 331.0 on a claim over the three-year index period. A total of 865 cases met this criterion. Results from regression models are presented below in eTable 5:

© 2019 Chapman BP et al. *JAMA Psychiatry.*

eTable 5. Associations Between Personality Traits and CMS AD Classification

	Sociabil-Ity Model	Social Sensitivity Model	Impuls-Ivity Model	Vigor Model	Calm Model	Tidiness Model	Culture Model	Leader-Ship Model	Self-Confidence Model	Maturity Model
Sociability	0.90**									
	0.84, 0.96									
Social Sensitivity		0.92*								
		0.86, 0.98								
Impulsivity			1.03							
			0.97, 1.10							
Vigor				0.90**						
				0.84, 0.96						
Calm					0.93*					
					0.86, 0.99					
Tidiness						0.96				
						0.89, 1.02				
Culture							0.91**			
							0.85, 0.98			
Leadership								0.96		
								0.89, 1.02		
Self-Confidence									0.90**	
									0.84, 0.97	
Maturity										0.91**
										0.85, 0.97
Sophomore	1.20	1.20	1.19	1.20	1.20	1.20	1.20	1.19	1.20	1.20
	0.97, 1.48	0.98, 1.48	0.97, 1.47	0.97, 1.47	0.98, 1.48	0.97, 1.48	0.98, 1.48	0.97, 1.47	0.97, 1.48	0.98, 1.48
Junior	1.62***	1.64***	1.60***	1.61***	1.63***	1.62***	1.63***	1.61***	1.62***	1.64***
	1.32, 1.98	1.34, 2.01	1.31, 1.96	1.31, 1.97	1.33, 2.00	1.32, 1.98	1.33, 2.00	1.31,- 1.97	1.32, 1.99	1.34, 2.00
Senior	1.98***	2.01***	1.95***	1.96***	2.00***	1.98***	2.00***	1.96***	1.99***	2.01***
	1.63, 2.4	1.65, 2.44	1.60, 2.37	1.62, 2.38	1.65, 2.44	1.62, 2.40	1.65, 2.44	1.62, 2.38	1.64, 2.42	1.66, 2.44
Female	1.14	1.14	1.14	1.14	1.14	1.14	1.14	1.14	1.14	1.14
	0.99, 1.30	1.00, 1.30	1.00, 1.30	0.99, 1.30	0.99, 1.30	1.00, 1.30	1.00, 1.30	0.99, 1.30	0.99, 1.30	1.00, 1.30
African American	1.97***	2.00***	1.99***	1.99***	2.01***	2.00***	2.04***	2.03***	2.03***	2.00***
	1.50, 2.58	1.52, 2.63	1.51, 2.62	1.51, 2.61	1.53, 2.64	1.52, 2.63	1.55, 2.68	1.54, 2.67	1.54, 2.66	1.52, 2.63
Other Minority	0.75	0.76	0.76	0.74	0.76	0.76	0.76	0.76	0.76	0.75
	0.46, 1.18	0.48, 1.201	0.48, 1.20	0.47, 1.18	0.48, 1.19	0.48, 1.20	0.48, 1.19	0.48, 1.20	0.48, 1.20	0.48, 1.19
Missing Race	0.65	0.66	0.66	0.66	0.66	0.66	0.66	0.66	0.66	0.67
	0.21, 2.02	0.21, 2.06	0.21 - 2.07	0.21 - 2.05	0.21, 2.07	0.21, 2.07	0.21, 2.06	0.21, 2.06	0.21, 2.07	0.22, 2.10

© 2019 Chapman BP et al. *JAMA Psychiatry*.

SES	0.95	0.95	0.93	0.95	0.95	0.94	0.96	0.94	0.95	0.95
	0.88, 1.02	0.88, 1.02	0.87 - 1.00	0.88 - 1.02	0.88, 1.02	0.88, 1.01	0.89, 1.03	0.88, 1.02	0.88, 1.02	0.88, 1.02

Notes: N = 82,232. * = p < .05, ** = p < .01, *** = p < .001. Model with interactions showed interaction terms HR 95% CI of .95 .89, 1.02 for Calm and .94 .88, 1.01 for Maturity, compared to .94 and .93, respectively, for ADRD classification.

© 2019 Chapman BP et al. *JAMA Psychiatry*.

eAppendix 3. Sensitivity Analysis

3A: Selection Models

The Medicare subsample may differ in race and SES from the 1960 baseline probability sample due to early mortality, differential use of Part A or B data (i.e., Part C only), or many other factors that might or might not affect the observed associations. To examine this, we compared relative risk regression models (i.e., Poisson distribution with robust variance estimate[2]) in the Medicare sample with Heckman selection models in which the selection equation is a probit model and the main regression takes the same relative risk specification. Covariate control is identical to the main models, and selection equation for each model contains SES, race, and the personality trait appearing in the main equation.

Results for the personality traits are presented in the following table, in the form of log relative risks, standard errors, and 95% confidence models in the two different types of models. Minimal changes in estimate occur. Thus, differences between the analytic sample and broader baseline, which primarily involve SES and race, do not appear to lead to substantially different results when considered in selection models.

© 2019 Chapman BP et al. *JAMA Psychiatry.*

eTable 6. Comparison of Medicare Analytic Sample Model vs Selection Model Results

	Analytic Sample Model	Sample Selection Model
	Beta (SE) 95% CI	Beta (SE) 95% CI
Sociability	-0.04*	-0.04*
	(0.02)	(0.02)
	-0.08, -0.00	-0.08, -0.00
Social Sensitivity	0.01	0.01
	(0.02)	(0.02)
	-0.03, 0.05	-0.03, 0.05
Impulsivity	0.04*	0.04*
	(0.02)	(0.02)
	0.00, 0.08	0.00, 0.08
Vigor	-0.07***	-0.07***
	(0.02)	(0.02)
	-0.11, -0.03	-0.11, -0.03
Calm	-0.05**	-0.05**
	(0.02)	(0.02)
	-0.09, -0.01	-0.09, -0.02
Tidiness	-0.03	-0.03
	(0.02)	(0.02)
	-0.07, 0.00	-0.07, 0.00
Culture	-0.03	-0.03
	(0.02)	(0.02)
	-0.07, 0.01	-0.07, 0.01
Leadership	0.02	0.02
	(0.02)	(0.02)
	-0.02, 0.05	-0.02, 0.05
Self-Confidence	-0.06**	-0.06**
	(0.02)	(0.02)
	-0.09, -0.02	-0.10, -0.02
Maturity	-0.04*	-0.04*
	(0.02)	(0.02)
	-0.08, -0.01	-0.08, -0.00
Calm	-0.04*	-0.04*
	(0.02)	(0.02)
	-0.08, -0.00	-0.08, -0.00

Notes. * = p < .05, ** = p < .01, *** = p < .001. Model with interactions showed interaction terms log RR (SE) [95% CI] for calm of -.07 (.02) [-.11, -.02] in analytic sample model vs. -.07 (.00) [-.07, -.07] in selection model, and for calm, -.07 (.02) [-.11, .02] in analytic sample model vs. -.06 (.00) [-.06,-.06] in selection model for maturity. Standard errors are less than .004 for these terms.

© 2019 Chapman BP et al. *JAMA Psychiatry.*

3B: Diagnostic Sensitivity Simulation

We conducted Monte Carlo Sensitivity Analyses for misclassification[3] to probe the potential impact of changes in sensitivity of the CMS diagnostic algorithm for ADRD, following a recent paper of similar study design[4]. These analyses simulated relative risk regression models with the same sample size and event rate observed in the data, and used a relative risk estimate of .9 for an exposure with a standard normal distribution, corresponding the covariate-adjusted relative risk calm and maturity at +1 SD SES.

Non-differential misclassification would be expected to produce little change in point estimates, but to increase standard errors. For this simulation, we drew sensitivity estimates from a uniform distribution varying + / - 5% around the published sensitivity estimate of .86. As in prior work[4], specificity was held fixed to prevent epidemiologically implausible changes in the rare event rate i.e., a 3% dementia prevalence at age 70 exploding to 30% with 10% variations in specificity. 1000 simulations were run for this condition, and eFigure 2 shows the results.

As can be seen from eFigure 2, the impact of non-differential misclassification is primarily to increase the standard error of the estimate. For the purposes of inference, at the lowest examined sensitivity .81, the highest standard error of .022 is observed, yielding a z-statistic of -.1054/.022 = -.4.79, yielding a p-value below .001.

Next, we examined the potential impact of differential misclassification according to levels of calm or maturity in high school. There is no definitive way to know what form this sort of differential sensitivity would take, since several scenarios can be envisioned. For instance, students who were less calm in high school may be more likely to experience health anxiety or other problems causing them to come to the attention of the medical system, where a dementia might be diagnosed. The opposite could occur if people who experienced health anxiety were more likely to avoid the medical system as a form of coping. Similarly, more mature high school students might exercise greater responsibility in pursuing regular health care later in life, where a dementia could be detected. Alternatively, a high level of responsibility might lead them into caregiving roles for others, causing them to neglect their own health care needs in favor of those of their charges.

To encompass this range of possibilities, we examined the impact of a sensitivity drawn from a uniform distribution lower by up to 5% in those in the bottom half of a trait (i.e., sensitivities of .81-.86) while simultaneously higher by the same amount in those in the top half the trait (i.e., sensitivities of .86. - .91). Thus, up to a 10% range in specificity was again assessed, but this time it varied systematically according to the trait.

Results indicated that if sensitivity is assumed to be systematically higher in those scoring high on a protective trait such as calm and maturity, the protective association is overestimated. This is illustrated in the top portion of eFigure 3 which shows the log relative risk attenuating as sensitivity is assumed to be increasingly greater among those high vs. low in a protective trait. At the maximum discrepancy of 10%—a sensitivity of .81 among those low in calm and .91 among those high in calm, for instance—the protective association is diminished to a log relative risk of .94.

The bottom portion of eFigure 3 shows the impact on the relative risk is the opposite type of misclassification that is assumed to occur—that is, sensitivity is higher among those higher in calm or maturity. In this case, the observed relative risk of .9 is an underestimate of the true relative risk. At a maximum of 10% difference—that is, a sensitivity of .91 among those low in calm, and .81 among those high in calm, for instance—the true protective association is actually greater, with a relative risk of approximately .87. In both scenarios standard error is not systematically affected. Thus, the simulations results of differential sensitivity by levels of high school personality reveal that, depending on the assumptions, the observed relative risks of roughly .9 for calm and maturity at higher SES may be either underestimates or overestimates. The direction depends on whether one believes persons who were calm and mature as adolescents in 1960 are more or less likely to seek care leading to ADRD diagnoses in the index period.

© 2019 Chapman BP et al. *JAMA Psychiatry.*

eFigure 2. Impact of Nondifferential Misclassification on Log Relative Risk and Standard Error

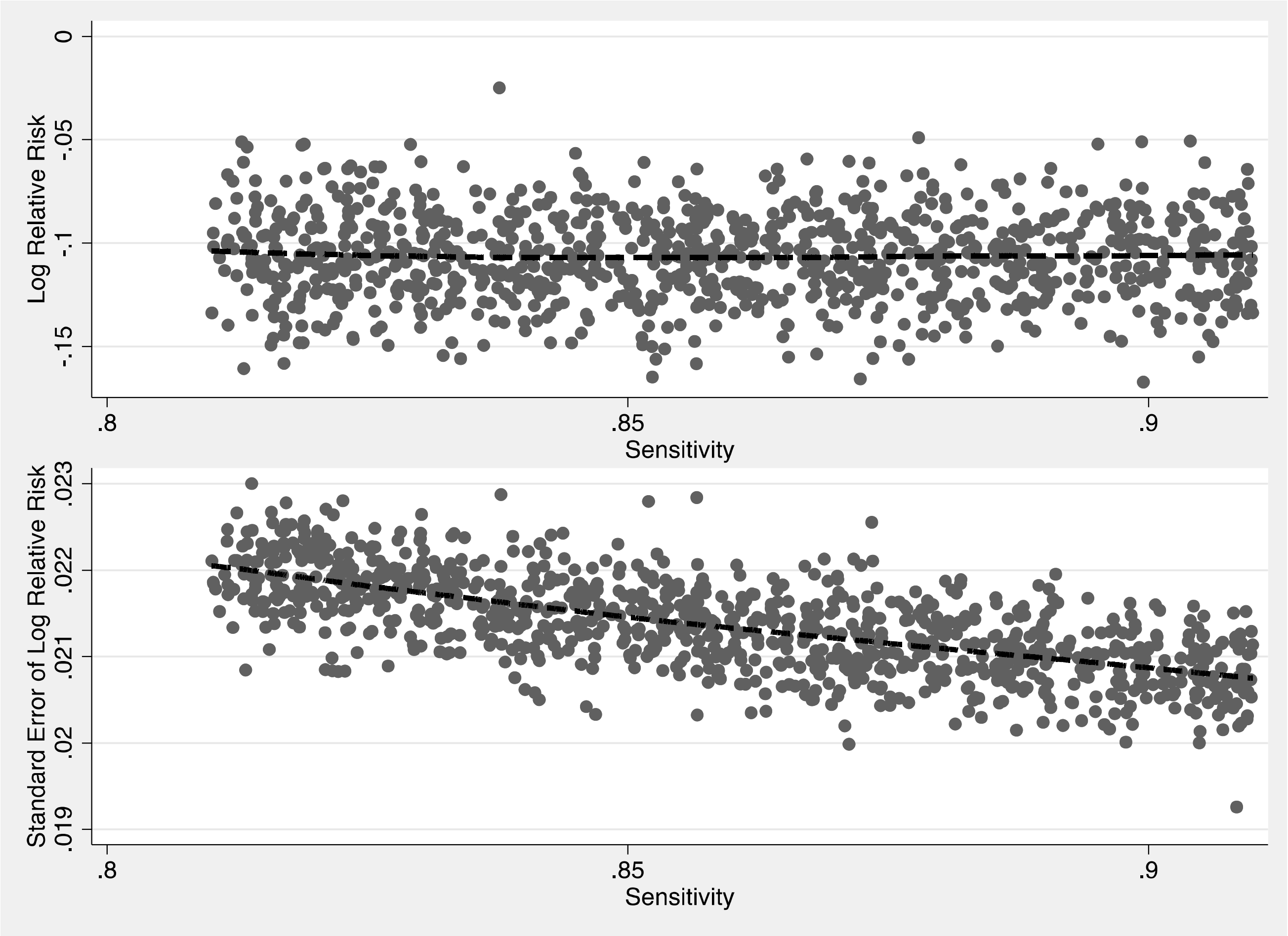

eFigure 2 caption: Results of 1000 simulations of the impact of randomly varying sensitivity on log relative risk corresponding to that seen for calm and maturity at +1 SD SES and the standard error of that log relative risk.

© 2019 Chapman BP et al. *JAMA Psychiatry.*

eFigure 3. Impact of Differential Misclassification on Log Relative Risks

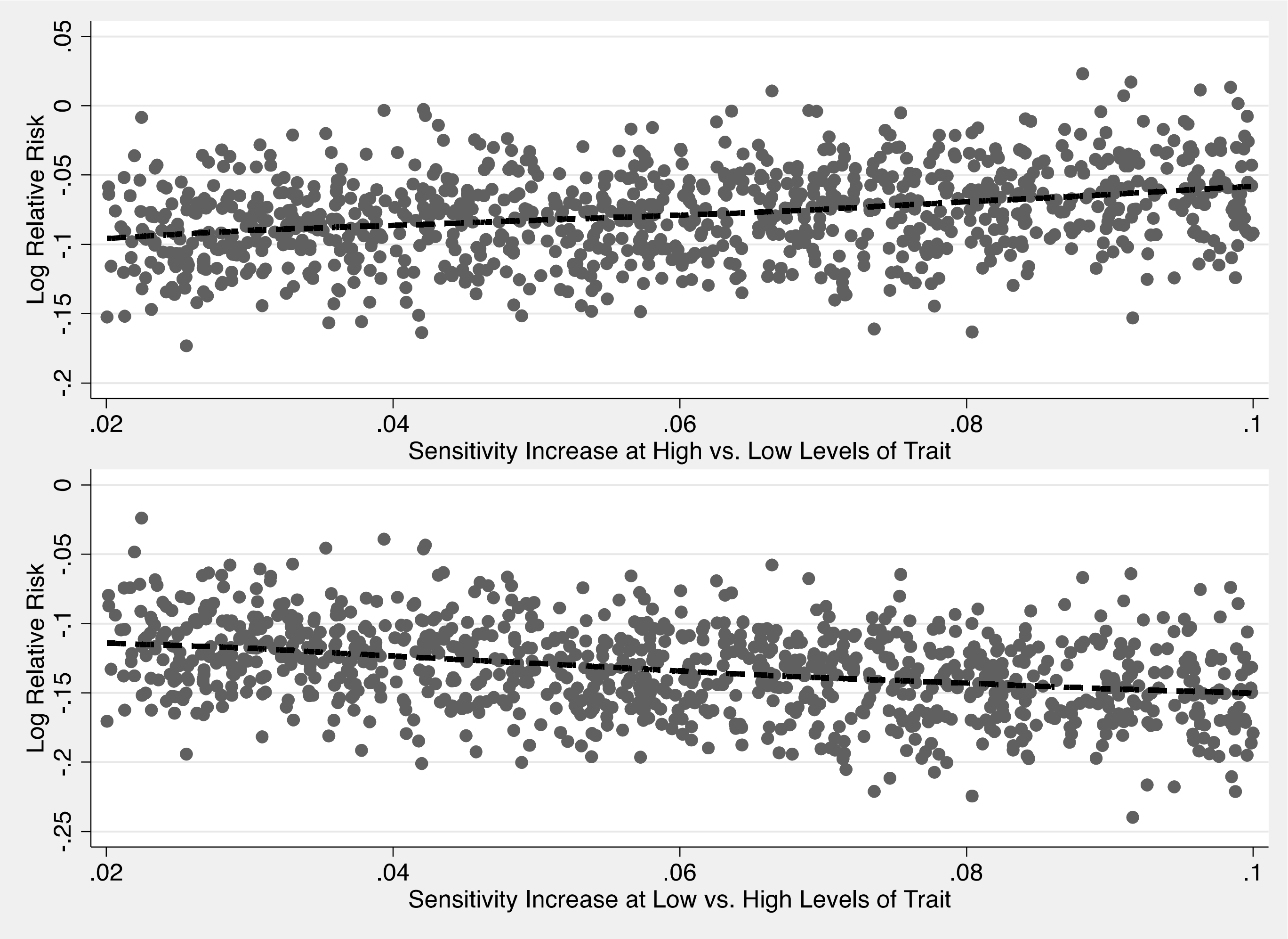

eFigure 3 caption: Results of 1000 simulations of the impact of sensitivity varying systematically by level of personality trait on log relative risk corresponding to that seen for calm and maturity at +1 SD SES and the standard error of that log relative risk. The top portion shows results from the condition in which sensitivity is higher among those high in the trait, while the bottom portion shows the results from the condition in which sensitivity is higher among those low in the trait

© 2019 Chapman BP et al. *JAMA Psychiatry.*

3C: High School Personality Traits and Service Use in the Index Period

To further study this question, we examined whether any 1960 traits were associated with the total amount of fee-for-service outpatient visits across 2012-2013 (the years for which claims data are available for this sample). These included outpatient visits to general practitioners, geriatricians, family medicine physicians as well as specialists liable to render such diagnoses (psychiatrists and neurologists). Because the hypothetical process involves utilizing services and receiving a dementia diagnosis as a result, this analysis focused on those who had not yet received a diagnosis. Service use would likely be elevated after a diagnosis as a result of the diagnosis, rather than high school personality traits. Number of visits was modeled as a negative binomial outcome, to account for clustering within high utilizers, and employed the same set of covariates as the primary models for dementia. ETable 4 presents the results.

Neither vigor, calm, nor maturity were associated with greater service use in these analyses. With no management of Type I error due to the exploratory nature of the analysis, social sensitivity and culture showed nominally significant associations, whereby persons higher on these traits in high school used services during 2012-2013 at a greater rate (rate ratio for +1 SD social sensitivity = 1.05, 95% CI = 1.00, 1.09; rate ratio for +1 SD culture = 1.06, 95% CI = 1.02, 1.10). Sex was the primary driver of service use, with women using more services than men (rate ratio = 1.22, 95% CI= 1.13, 1.32). Thus, available data do not suggest that the high school traits associated with later dementia are associated with greater use of services where diagnoses might be received.

© 2019 Chapman BP et al. *JAMA Psychiatry.*

eTable 7. High School Personality Traits and Service Use Across Medicare Index Period

	Sociabil-Ity Model	Social Sensitivity Model	Impuls-Ivity Model	Vigor Model	Calm Model	Tidiness Model	Culture Model	Leader-Ship Model	Self-Confidence Model	Maturity Model
Sociability	0.02									
	-0.02, 0.06									
Social Sensitivity		0.04*								
		0.00, 0.08								
Impulsivity			0.02							
			-0.03, 0.06							
Vigor				0.00						
				-0.04, 0.04						
Calm					0.00					
					-0.04, 0.04					
Tidiness						0.04				
						0.00, 0.008				
Culture							0.06**			
							0.02, 0.10			
Leadership								0.04		
								-0.00, 0.08		
Self-Confidence									-0.02	
									-0.05, 0.02	
Maturity										0.01
										-0.03, 0.05
Sophomore	0.03	0.03	0.03	0.03	0.03	0.03	0.03	0.03	0.03	0.03
	-0.08, 0.14	-0.08, 0.14	-0.08, 0.14	-0.08, 0.14	-0.08, 0.14	-0.08, 0.14	-0.08, 0.14	-0.08, 0.14	-0.08, 0.14	-0.08, 0.14
Junior	0.09	0.09	0.09	0.10	0.10	0.09	0.09	0.10	0.10	0.09
	-0.02, 0.21	-0.02, 0.20	-0.02, 0.21	-0.02, 0.21	-0.02, 0.21	-0.02, 0.20	-0.02, 0.20	-0.02, 0.21	-0.02, 0.21	-0.02, 0.21
Senior	0.19**	0.18**	0.19**	0.19**	0.19**	0.18**	0.18**	0.18**	0.19***	0.18**
	0.07, 0.30	0.06, 0.30	0.07, 0.30	0.08, 0.30	0.08, 0.30	0.07, 0.30	0.07, 0.29	0.07, 0.30	0.08, 0.30	0.07, 0.30
Female	0.20***	0.20***	0.20***	0.20***	0.20***	0.20***	0.20***	0.20***	0.20***	0.20***
	0.12, 0.28	0.12, 0.28	0.12, 0.28	0.12, 0.28	0.12, 0.28	0.12, 0.28	0.12, 0.28	0.12, 0.28	0.12, 0.28	0.12, 0.28
African American	-0.02	-0.03	-0.02	-0.02	-0.02	-0.03	-0.03	-0.04	-0.02	-0.02
	-0.24, 0.20	-0.25, 0.20	-0.25, 0.20	-0.25, 0.20	-0.25, 0.20	-0.25, 0.20	-0.26, 0.19	-0.26, 0.19	-0.25, 0.20	-0.25, 0.20
Other Minority	0.10	0.10	0.10	0.10	0.10	0.10	0.10	0.10	0.10	0.10
	-0.13, 0.34	-0.13, 0.33	-0.13, 0.34	-0.14, 0.33	-0.14, 0.33	-0.14, 0.33	-0.13, 0.34	-0.14, 0.33	-0.14, 0.33	-0.14, 0.33
Missing Race	0.10	0.10	0.11	0.10	0.10	0.10	0.10	0.10	0.10	0.10
	-0.35, 0.56	-0.36, 0.56	-0.35, 0.57	-0.36, 0.56	-0.36, 0.56	-0.36, 0.56	-0.34, 0.57	-0.36, 0.56	-0.36, 0.56	-0.36, 0.56

© 2019 Chapman BP et al. *JAMA Psychiatry*.

SES	0.00	0.00	0.01	0.01	0.01	0.00	0.00	0.00	0.01	0.01
	-0.04, 0.05	-0.04, 0.04	-0.04, 0.04	-0.04 - 0.05	-0.03, 0.05	-0.04, 0.04	-0.05, 0.04	-0.04, 0.04	-0.03, 0.05	-0.04, 0.05
Constant	-0.92***	-0.92***	-0.92***	-0.92***	-0.92***	-0.92***	-0.92***	-0.92***	-0.92***	-0.92***
	-1.01, -0.83	-1.01, -0.83	-1.01, -0.83	-1.01 - -0.84	-1.01, -0.84	-1.01, -0.83	-1.01, -0.83	-1.01, -0.83	-1.01, -0.84	-1.01, -0.83

Notes. Results from negative binomial regression models. Top portion of cell is beta, bottom portion is 95% Confidence Interval. N=78,818. * = $p < .05$, ** = $p < .01$, *** = $p < .001$.

© 2019 Chapman BP et al. *JAMA Psychiatry*.

eReferences

1. Jarosek S. Identifying Medicare Managed Care Beneficiaries from the Master Beneficiary Summary or Denominator Files. 2018; https://www.resdac.org/articles/identifying-medicare-managed-care-beneficiaries-master-beneficiary-summary-or-denominator. Accessed January 4, 2019.
2. Zou G. A modified poisson regression approach to prospective studies with binary data. *Am J Epidemiol.* 2004;159(7):702-706.
3. Lash TL, Fox, M.P., & Fink, K. *Applying Quantitative Bias Analysis to Epidemiologic Data.* New York: Springer; 2009.
4. Gradus JL, Horváth-Puhó E, Lash TL, et al. Stress Disorders and Dementia in the Danish Population. *Am J Epidemiol.* 2018.

© 2019 Chapman BP et al. *JAMA Psychiatry.*
